# Comparison of two caries prevention programs among Thai kindergarten: a randomized controlled trial

**DOI:** 10.1186/s12903-020-01107-5

**Published:** 2020-04-19

**Authors:** Pagaporn Pisarnturakit, Palinee Detsomboonrat

**Affiliations:** Department of Community Dentistry, Faculty of Dentistry, Chulalongkorn University, 34 Henry Dunant Road, Patumwan, Bangkok, 10330 Thailand

**Keywords:** Fluoride, Kindergarten, Prevention programs, Randomized controlled trial, Risk assessment, Toothbrushing

## Abstract

**Background:**

Intensified preventive regimen based on a ‘high-risk’ approach has been proposed instead the routine prevention that is generally given to the whole population. The effectiveness of these regimens may still be an issue. Therefore, the aim of this study was to compare two preventive programs carried out in a Public School for kindergarten children.

**Methods:**

The data from clinical examinations were used to assess the caries risk for 121 children. Children with at least 2 carious lesions were considered as high risk for dental caries development. These children were randomized into two groups. Half (High risk basic-HRB group) were provided the basic prevention regimen (oral-hygiene instruction and hands-on brushing practice for teachers and caregivers, daytime tooth brushing supervised by teachers at least once a week, newly erupted first permanent molar sealant, provision of toothbrush, fluoride-containing dentifrice, and a guidebook), which was also given to low-risk children (Low risk basic-LRB group). The other half (High risk intensive-HRI group) were additionally given an intensified preventive regimen (F-varnish application, primary molar sealant, and silver diamine fluoride (SDF) application on carious lesions). Clinical examinations were performed semiannually to determine the dmfs caries increment of the three groups.

**Results:**

The 89 children completed the 24-month examination were 3- to 5-year-old with 19, 35, and 35 children in the LRB, HRB, and HRI group, respectively. The new caries development at 24 months of the HRB group (75%) was higher than that of the HRI group (65.7%) and the LRB group (21.1%). One-way analysis of variance (ANOVA) indicated no significant differences of caries increment between the HRB and HRI groups at the end of our study (*p* = 0.709).

**Conclusions:**

The negligible difference in caries increment between the HRI and HRB groups implies that intensified prevention produced minimal additional benefit. Offering all children only basic prevention could have obtained virtually the same preventive effect with substantially less effort and lower cost.

**Trial registration:**

Thai Clinical Trials Registry (TCTR), TCTR20180124001. Registered 24 January 2018 - Retrospectively registered.

## Background

Although there have been dramatic declines in the dental caries burden in the last 20 years in several industrialized countries, the dental caries experience in Thailand has remained approximately unchanged for 30 years, particularly in 3–5-year-old Thai children [1]. These data indicated the need for an effective caries control regimen among young children. Some studies argued that prevention strategies that focused solely on those at higher risk have less impact compared with strategies that focused on the whole population, because the majority of dental caries will occur among those not at high risk [2, 3]. However, strategies that impact whole populations frequently require action beyond the health care sector and are politically more difficult to achieve.

Although caries preventive measures, such as early detection, risk assessment, supervised tooth brushing, sealant application, and topical fluoride application have proven effective in clinical trials [4–8], there is little investigation of the high-risk regimen in real-life conditions, especially for young children. In our study, the basic preventive regimen including supervised tooth brushing was given to the whole population and an intensified regimen of fluoride varnish, sealant, and silver diamine fluoride application comprised the intensified preventive regimen. Various effective preventive measures have been separately implemented among the children with dental caries but there is little evidence of their effect when they were applied together. Moreover, it wondered that high cost and time consume procedure would give high result or not. Therefore, the aim of this study was to evaluate whether the intensified preventive regimen was more effective compared with the basic preventive regimen for decreasing caries development in young children.

## Methods

This study was registered in the Registry of Clinical Trials run by the Thai Clinical Trials Registry (TCTR) (TCTR20180124001). Ethical approval was obtained from the institute’s committee on human research of the Faculty of Dentistry, Chulalongkorn University (HREC_DCU 2017–084). Written informed consent was obtained from the parents or guardians. The recruitment period started in September 2017 and ended in January 2018. The last follow-up examination was performed in December 2019.

### Study design

This 24-month study was conducted in 128 kindergarten children in Naiyananon-Anusorn School, Bangkok. Healthy children aged 3- to 5-years-old were recruited. The parents of the children received a letter in which the study design was explained and they were asked to give their informed consent. The parents of 2 children refused to give their permission and 5 children were absent at the first examination. A total of 7 children were excluded from the study. Therefore, 121 children were assessed the caries risk and information about their socio-demographic background, habits related to oral health, perception related to dental caries, and perceived self-efficacy in performing tooth brushing was obtained using a self-administered questionnaire that was given to the primary caregiver. Comparing the basic preventive regimen and the intensified preventive regimen by measuring the incremental caries would not fare if they have different risk of dental caries. Therefore, caries risk was assessed as high risk and low risk using data from the clinical examinations. Two dentists conducted the oral examination with blinding the child’s assigned group under mobile dental units at the school. Dental caries and oral hygiene status were examined under the mobile unit light using a ball-ended probe and a mouth mirror. The classification of the dental findings followed the WHO criteria for the dmft/dmfs indices and the visible plaque score (VPI) was determined according to the Simplified Oral hygiene Index (S-OHI). This index assesses the amount of debris found on the buccal or lingual surface of each of the selected teeth [9]. The inter- and intra-examiner reliabilities were measured using Cohen’s kappa by examining 10% of the children twice by the two dentists in each survey for inter-examiner and twice in each dentist for intra-examiner. The kappa values for the inter- and intra-examiner reliability were 0.82–0.88.

**Fig. 1:**
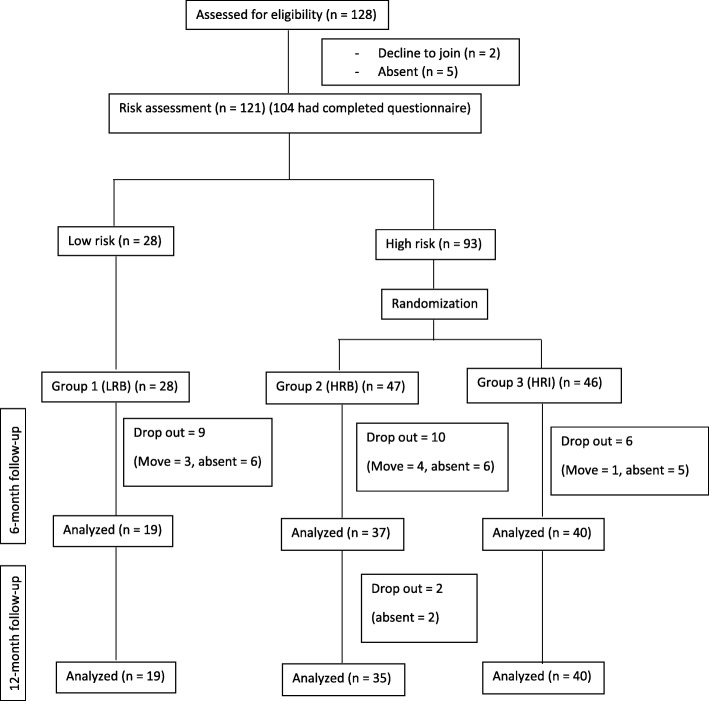
Subject allocation into the different groups

To assess caries risk for each individual, number of dentinal carious lesion were examined. If at least two carious teeth were present, the child was categorized as high-risk. The remaining children were assigned to the low-risk group. Based on this, 93 children had caries risk that was considered high (High risk in Fig. 1). The children who were regarded as being at high risk were randomly allocated by a research coordinator into the High Risk Intensive intervention (HRI) group or the High Risk basic intervention (HRB) group. The sequences were then sealed in an opaque envelop, and the enveloped were numbered. The research coordinator assigned children into two groups after opening the sealed envelope and reviewing the allocation sequences. The low risk children received the basic preventive regimen (LRB). Follow-up examinations using dmft/dmfs indices and caries increment were performed at 6, 12 and 24 months as described above by the same two calibrated dentists for whom the treatment group allocation was masked. Subject allocation into the different groups is shown in Fig. 1.

### Study regimen

The basic preventive regimen for all groups comprised of 1) Instruction of good oral hygiene practices, appropriate diet and hands-on brushing practice was given to caregivers and kindergarten-teachers on parents meeting day at the school by two dentists, 2) Supervised tooth brushing by 10 kindergarten-teachers at daytime brushing and tooth brushing by the teacher for each child once a week, 3) Application of sealant to newly erupted first permanent molars by two dentists, and (4) Each caregiver was provided a toothbrush, fluoride-containing dentifrice, and a guidebook on the tooth brushing method.

The additional professional measures in the intensified preventive regimen given to the HRI group consisted of 1) F-varnish (3 M™ Vanish™, Canada) was applied every 6 months on the tooth surfaces, 2) Sealants were also placed on primary molars with deep pits and fissures, and 3) 38% silver diamine fluoride (SDF) solution (Topamine™ Dental life, Australia) was applied every 6 months on any carious lesions.

### Sample-size calculation

A power analysis using the G*power computer program (Faul & Erdfelder, 1998) indicated that a total sample of 62 people for HRI and HRB group would be needed to detect moderate effects (effect size = 0.3) with 80% power using ANOVA between means with alpha at 0.05. The required sample size calculated using the equation for ANOVA repeated measurement was 31 children for each group (HRB and HRI group). With an anticipated 20% dropout rate, 38 children for HRB and HRI group were needed at baseline. Moreover, this study based on the following rules of thumb [10] that the minimum ratio of observations to variables is 10:1 for a logistic regression analysis.

### Statistical analysis

The data were statistically analyzed using the SPSS 22.0 for Windows (IBM Corp., Armonk, New York, USA) program. Kappa statistics was adopted to determine the inter- and intra-examiner reliability in caries diagnosis and oral hygiene assessment. The Chi-square test and one-way analysis of variance (ANOVA) were used to compare the baseline information of the study children among three groups. The student’s *t*-test was used to evaluate the statistical significance of the mean difference in dmfs and VPI between subjects who completed the follow-up and subjects who were lost to follow-up and a post hoc test following ANOVA was used to compare differences among three groups for pairwise comparisons after intervention. A multiple logistic regression model was constructed to identify the variables associated with binary outcome (0 for “No newly developed caries”, 1 for “Having newly developed caries”). The variables associated with newly developed caries in the univariate analysis were eligible for entry into multiple logistic regression models if they were significantly associated at *p* < 0.20. Children’s gender, dmft score, risk group, perceived susceptibility, perceived severity to dental caries were chosen and entered into the baseline model. Because treatment group was the main interest factor of the study, it was forced to stay in the final model. Odds ratios and 95% confidence intervals (CI) were calculated. Estimated coefficients and their standard errors (SEs) were calculated using the method of maximum likelihood. Variables were eliminated from the model one at a time based on likelihood ratio tests. When all nonsignificant (*p* > 0.05) variables had been eliminated from the multivariate model, calibration was assessed using the Hosmer-Lemeshow goodness-of-fit test. This test evaluates the degree of correspondence between a model’s estimated probability of new caries and the actual new caries of the children spanning the entire range of probability.

## Results

A total of 121 kindergarten children (67 boys, 54 girls) with 28, 47, and 46 children in the LRB, HRB, and HRI group, respectively, were recruited in this trial. At baseline, the mean (standard deviation (SD)) caregiver age was 36.6 (9.2) years. The children’s mean (SD) decayed, missing, and filled score was 5.68 (5.07) teeth and 13.04 (15.37) surfaces, respectively. Plaque was found on the children’s tooth surfaces with a visible plaque index score (VPI = 1.11). There was no significant difference between the children in the three study groups regarding their demographic background, oral health–related behaviors, perceptions related to dental caries, and tooth brushing self-efficacy (χ^2^ test and ANOVA at *p* > 0.05) (Table 1). Clinically, the children whose risk was considered high (HRI and HRB) had markedly higher baseline dmft/dmfs scores compared with those for whom the risk was considered low (LRB). However, there was no significant difference between the two high-risk (HRI and HRB) groups in baseline dmft/dmfs (*p* = 0.992).

**Table 1:** Children’s demographic background, clinical characteristics, oral health–related behaviors, perception and self-efficacy

Group	LRB	HRB	HRI	*p* value^a^
	N (%)	N (%)	N (%)	
Demographic background				
Child’s sex: boy	19 (67.9%)	23 (48.9%)	25 (54.3%)	0.276
Caregiver age (years): Mean (SD)	36.5 (8.3)	36.3 (8.5)	37.5 (10.3)	0.699^b^
Relationship to child				0.066
Father or mother	17 (81.0%)	39 (92.9%)	31 (73.8%)	
Grandparents/Relatives	4 (19.0%)	3 (7.1%)	11 (26.2%)	
Education level				0.931
Primary school or less	5 (26.3%)	10 (25.6%)	12 (29.3%)	
High school or more	14 (73.7%)	29 (74.4%)	29 (70.7%)	
Occupation level (mother or father)				0.159
More stable occupation	0 (0%)	4 (9.5%)	1 (2.4%)	
Less stable occupation	21 (100%)	38 (90.5%)	41 (97.6%)	
Monthly family income in Baht ($)				0.554
Below 10,000 ($318.82)	5 (27.8%)	12 (29.3%)	6 (14.6%)	
10,001–30,000 ($318.82–956.48)	10 (55.6%)	20 (48.8%)	25 (61.0%)	
Above 30,000 ($956.48)	3 (16.7%)	9 (22.0%)	10 (24.4%)	
Prior experience with children: yes	19 (95.0%)	37 (92.5%)	38 (92.7%)	0.930
Clinical characteristics Mean (SD)				
dmft score	0.79 (3.39)	7.30 (4.63)	7.00 (4.51)	< 0.001^b^
dmfs score	1.96 (8.86)	16.49 (14.80)	16.26 (16.03)	< 0.001^b^
VPI score	0.99 (0.74)	1.31 (0.81)	0.96 (0.55)	0.063^b^
Oral health behaviors				
Brushing at first tooth eruption				0.188
Yes	8 (38.1%)	26 (61.9%)	21 (50.0%)	
No	13 (61.9%)	16 (38.1%)	21 (50.0%)	
Frequency of brushing				0.169
2 time or less / week	3 (14.3%)	0 (0%)	3 (7.3%)	
3–5 times /week	1 (4.8%)	6 (14.3%)	5 (12.2%)	
5 times or more /week	17 (81.0%)	36 (85.7%)	33 (80.5%)	
Frequency of snacking between meals				0.241
Never or 1 time / day	9 (42.9%)	14 (34.1%)	15 (35.7%)	
2 times /day	6 (28.6%)	11 (26.8%)	19 (45.2%)	
3 times or more /day	6 (28.6%)	16 (39.0%)	8 (19.0%)	
Perception related to dental caries (Mean (SD))				
Perceived susceptibility	18.4 (7.3)	18.0 (6.8)	17.0 (5.5)	0.641^b^
Perceived severity	24.3 (3.1)	24.2 (3.6)	23.9 (4.9)	0.904^b^
Perceived benefits	21.2 (2.2)	21.9 (2.3)	21.0 (4.2)	0.504^b^
Perceived barriers	11.5 (3.9)	12.5 (3.3)	12.3 (4.4)	0.648^b^
Tooth brushing self-efficacy (Mean (SD))	7.8 (1.4)	7.7 (1.6)	7.9 (1.6)	0.816^b^

^a^χ^2^ test and ^b^ANOVA

A total of 96, 94 and 89 children were re-evaluated at 6-, 12-, 24-month follow-up. The overall dropout rate was 26.4% with 32.1, 25.5, and 23.9% in the LRB, HRB, and HRI group, respectively. Moving to another school and absent from school on the examination date were the reasons for leaving this study (Fig. 1). There were no significant differences in the demographic background, oral health–related behaviors, and clinical characteristics between the children who completed the study and those who were lost to follow-up (*p* > 0.05) [see Additional file 1]. At least one tooth of newly developed caries was found in 35.4% of total children at the 6-month follow-up and this percentage rose to 60.7% at the 24-month follow-up (Table 2). Caries increment highly increased over time in all groups, particularly in the children classified as high caries risk The percentage of new caries development in the HRB group increased from 45.9% (at 6 months) to 75% (at 24 months), similar to those in the HRI group demonstrated an increase from 40% (at 6 months) to 65.7% (at 24 months) (Table 2). Notably, the intensified preventive regimen intended for use in high caries children did not help to control new dental caries development. A total active lesion of 138 surfaces from 159 surfaces (86.8%) became arrested caries after SDF-treatment for HRI group.

**Table 2:** Sample distribution by new caries in 24 month according to risk group and intervention

Group	Intervention	No. of children				No. of children with new caries		
		Baseline	6 month	12 month	24 month	6 month (t)	12 month (t)	24 month (t)
LRB	standard	28	19	19	19	1 (5.3%)	2 (10.5%)	4 (21.1%)
HRB	standard	47	37	35	35	17 (45.9%)	15 (42.9%)	27 (75%)
HRI	Intensive	46	40	40	35	16 (40%)	20 (50%)	23 (65.7%)
**Total**		**121**	**96**	**94**	**89**	**34 (35.4%)**	**37 (39.4%)**	**54 (60.7%)**

The increment dmfs/DMFS and increment dmft/DMFT at 6-, 12-, 24-month follow-up were significantly higher in the two high-risk groups compared with the low-risk group. However, there were no significant differences between the HRB and HRI groups in caries increment for pairwise comparisons at 6-, 12-, 24-month-follow up. For the children in the HRB group, the total increment (6.75 surfaces) was higher than those in the HRI group (5.91 surfaces). However, there was no significant difference between the HRB and HRI groups at the end of our study (*p* = 0.709) (Table 3).

**Table 3:** Caries increment of LRB, HRB and HRI group at 6, 12 and 24 months

	Caries severity	Group	Mean (SD)	ANOVA	Games-Howell test		
				*p*-value	pairwise comparison		*p*-value
6 m	Increment dmfs/DMFS	LRB	0.05 (0.23)	< 0.001*	LRB	HRB	0.001*
		HRB	2.52 (2.58)		LRB	HRI	0.002*
		HRI	2.83 (3.27)		HRB	HRI	0.824
	Increment dmft/DMFT	LRB	0.05 (0.23)	< 0.001*	LRB	HRB	< 0.001*
		HRB	0.76 (0.94)		LRB	HRI	< 0.001*
		HRI	0.93 (1.46)		HRB	HRI	0.893
12 m	Increment dmfs/DMFS	LRB	0.47 (1.17)	< 0.001*	LRB	HRB	< 0.001*
		HRB	5.09 (4.96)		LRB	HRI	< 0.001*
		HRI	4.45 (4.46)		HRB	HRI	0.818
	Increment dmft/DMFT	LRB	0.26 (0.65)	0.005*	LRB	HRB	0.038*
		HRB	1.03 (1.40)		LRB	HRI	0.011*
		HRI	0.95 (1.18)		HRB	HRI	0.999
24 m	Increment dmfs/DMFS	LRB	0.95 (2.12)	< 0.001*	LRB	HRB	< 0.001*
		HRB	6.75 (4.05)		LRB	HRI	< 0.001*
		HRI	5.91 (4.79)		HRB	HRI	0.709
	Increment dmft/DMFT	LRB	0.53 (1.12)	0.004*	LRB	HRB	0.008*
		HRB	1.67 (1.53)		LRB	HRI	0.012*
		HRI	1.57 (1.40)		HRB	HRI	0.960

Bivariate analyses of the independent variables are shown in Table 4. The variables of children’s gender, dmft score, risk group, perceived susceptibility, perceived severity to dental caries and treatment were selected for the multivariate analysis. The crude odd ratios (ORs) indicated that these variables were different between the children with absent and present new caries.

**Table 4:** Bivariate and Multivariable Logistic Regression for development of new caries at 24 months

Explanatory Variables	Crude OR	95% CI	*p* value	Adj. OR	95% CI	*p* value
Demographic background						
Age of caregiver (years)	1.014	(0.96, 1.07)	0.611			
Gender (reference: boy)	2.35	(0.97, 5.73)	0.060	2.14	(0.80, 5.73)	0.129
Main caretaker (reference: parent)						
Grandparent/Relatives	0.648	(0.20, 2.14)	0.475			
Caregiver’s occupation level (reference: more stable)						
Less stable	1.7	(0.57, 5.06)	0.334			
Caregiver’s education level (reference: High school)						
Primary school or less	0.955	(0.334, 2.72)	0.931			
Monthly family income (reference: above ฿30,000)						
Below ฿10,000($318.8)	0.69	(0.16, 3.04)	0.618			
฿10,001–฿30,000($318.8–956.5)	0.64	(0.18, 2.37)	0.506			
Clinical parameters at baseline						
dmft score	1.11	(1.01, 1.22)	0.025*	0.986	(0.88, 1.11)	0.809
Risk group (reference: low risk)	6.00	(2.04, 17.69)	0.001*	7.522	(1.66, 34.09)	0.009*
VPI score	1.53	(0.79, 2.94)	0.206			
Dental health–related habits at 12 mo						
Weekly frequency of toothbrushing (reference: 5 days or more)						
2 days or less / week	1.37	(0.23, 8.09)	0.727			
3–5 days /week	1.37	(0.23, 8.09)	0.727			
Daily frequency of snacking (reference: less than 2 times)						
2 times /day	0.66	(0.22, 2.00)	0.459			
3 times or more /day	2.35	(0.62, 8.86)	0.208			
Supervised toothbrushing (reference: yes)	1.55	(0.65, 3.69)	0.322			
Perception related to dental caries						
Perceived susceptibility	1.23	(1.05, 1.44)	0.007*	1.21	(1.00, 1.45)	0.048*
Perceived severity	1.11	(0.95, 1.31)	0.184	1.07	(0.88, 1.30)	0.486
Perceived benefits	1.17	(0.90, 1.52)	0.241			
Perceived barriers	1.08	(0.92, 1.27)	0.369			
Self efficacy to tooth brushing	1.05	(0.78, 1.41)	0.748			
Treatment (reference: basic prevention)						
Intensified prevention	1.42	(0.59, 3.44)	0.433	0.66	(0.21, 2.03)	0.471

The multiple logistic regression modeling resulted in a model containing 6 variables. Table 4 also presents the adjusted ORs, and the 95% confidence intervals (CIs) of the final model for new caries development. Risk group and perceived susceptibility still significantly affected new caries development after adjusting for other variables. Being in the high risk group had an OR of 7.52, signifying that the chance of new caries development would be 7.52 -fold higher than that of the low risk children. Moreover, for every 1 score increment in perceived susceptibility, odds of new caries increase 21%.

The results of the Hosmer-Lemeshow goodness-of-fit test indicated model good fit (*p* = 0.532). These results confirmed that there was no significant difference between the events observed and predicted by the model.

## Discussion

The results indicated that the children in the LRB group developed significantly fewer carious lesions compared with the high-risk children in both groups. These results indicate prediction of future caries in children by past caries experience, similar to those of several studies [8, 11–14]. At the end of the present study, there was no significant difference between the HRB and HRI groups in caries increment. Our results indicated that the additional intensified preventive regimen intended to control caries use in high caries populations did not further control dental caries, which is similar to previous studies showing intensifying prevention produced practically no additional benefit [15–17]. Hausen et al. found a no significant difference between intensified prevention and basic preventive programs in 12- to 13-year-old children. In contrast, there is a study indicated benefit from an intensified prevention program (fluoride varnish application, fissure sealing, and restorative therapy) in decreasing caries development [18]. These disparate results may be due to the absence of any oral hygiene instructions provision for the control groups while our basic preventive regimen group received interventions for achieving quality tooth brushing with no restorative therapy (oral health education with hand-on tooth brushing practice, supervised tooth brushing by their teachers during after-lunch brushing, and received a tooth brushing by their teacher at least once a week). The present study proposed a basic preventive regimen that differed from those of other studies, i.e. the provision of quality tooth brushing at least once a week. The provision of quality tooth brushing by the teacher once a week might be an important factor in the small dental caries increment among the three groups which was confirmed by several studies [19, 20]. In our study, every child brushed their teeth after-lunch under the supervision of their teacher every day, additionally, each child received tooth brushing by their teacher at least once a week. This strategy is practical as each teacher do the brushing for 5 children in 1 day during the assistant teacher supervised the children’s after-lunch tooth brushing. This practical strategy helped increase the quality of the child tooth brushing while reduce burden to the teacher. Provision of oral health education and hand-on tooth brushing practice to the teacher was arranged prior to the program commencement for emphasizing their crucial role and increasing the essential of their role. The session of oral health education emphasized the identification of plaque that causes carious lesions. They were also informed that white lesions could be reversed by a quality tooth brushing with fluoride toothpaste.

Although this study indicated no significant difference in caries increment between the two high-risk groups, the total increment in the HRB group was slightly higher than the HRI group at the end of study. One reason might be that the HRI group received additional professional preventive care at an early stage of caries progression, while the HRB group received the intensified tooth brushing regimen for preventing dental caries development. The potentially caries-preventive measures of sealant and fluoride varnish application used in HRI group protects against new caries development, while SDF is used for non-operative treatment for active caries. In this study, carious teeth became arrested caries after SDF-treatment for HRI group (86.8%). The active caries treated with SDF usually turns into arrested caries, which is normally brown or black, hard, glossy, and non-progressive. SDF inhibits tooth demineralization and promotes remineralization [21, 22]. However, this study did not alter the child eating habit which might affect the caries development. Moreover, the weekly quality tooth brushing with fluoride toothpaste by a teacher might be an effective potential caries prevention regimen. Then, the disappointingly low effect of the intensified prevention regimen was showed in the small difference between the high risk groups.

Factor associated with newly developed caries were identified by a multiple logistic regression model. The results, at baseline, indicated risk group is an important factor for new caries development among Thai kindergarten as children in high risk group have tendency to develop new caries more than others. These results re-emphasize the importance of ECC risk assessment among children.

Despite the rich data emerging from this study, limitations exist. The loss to follow up in our study was fairly high, especially in the low-risk group (32.1%). The number of lost children in the HRB group (25.5%) was higher than the HRI group (23.9%). High loss at follow up cannot be avoided in field studies.

## Conclusion

The negligible difference in caries increment between the HRI and HRB groups implies that intensified prevention produced minimal additional benefit. Offering all children only basic prevention could obtain virtually the same preventive effect with substantially less effort and lower cost. However, this strategy needs long term compliance from both teachers and parents to maintain the child’s good oral health. Tooth brushing should be performed on a regular basis and this habit should be instilled as a lifelong healthy behavior.

## Supplementary information

**Additional file 1.**

## Data Availability

All the datasets used and analyzed during the current study are available from the corresponding author on reasonable request.

## Abbreviations

HRB: High risk basic group
HRI: High risk intensive group
LRB: Low risk basic group
dmft / DMFT: Decayed, missing, filled teeth
dmfs / DMFS: Decayed, missing, filled surfaces
SDF: Silver diamine fluoride
VPI: Visible plaque score
S-OHI: Simplified Oral hygiene Index
