# Supplementary material for: Comparison of two caries prevention programs among Thai kindergarten: a randomized controlled trial
Source: BMC Oral Health. 2020 Apr 19;20:119. doi: 10.1186/s12903-020-01107-5 (PMC7168866; doi:10.1186/s12903-020-01107-5)
Supplement: Supplementary file 1 — Additional file 1. [file 12903_2020_1107_MOESM1_ESM.docx]

**Comparison of two caries prevention programs among Thai kindergarten: a randomized controlled trial**

Palinee Detsomboonrat and Pagaporn P. Pisarnturakit

**Appendix Table 1.** Demographic background, clinical characteristics and oral health–related behaviors between completed and lost subjects.

| Group | | | completed N (%) | lost N (%) | *p* value ^a^ |
| --- | --- | --- | --- | --- | --- |
| Demographic background | | |  |  |  |
|  | Child's gender | |  |  | 0.907 |
|  |  | Boy | 49 (55.1%) | 18 (56.3%) |  |
|  |  | Girl | 40 (44.9) | 15 (43.8%) |  |
|  | Age of caregiver(years): Mean (SD) | | 37.3 (9.2) | 36.1 (10.3) | 0.544 ^b^ |
|  | Relationship to child | |  |  | 0.552 |
|  |  | Father or mother | 69 (84.1%) | 23 (79.3%) |  |
|  |  | Grandparents/Relatives | 13 (15.9%) | 6 (20.7%) |  |
|  | Caregiver's education level | |  |  | 0.873 |
|  |  | Primary school or less | 21 (23.6%) | 8 (25.0%) |  |
|  |  | High school or more | 68 (76.4%) | 24 (75.0%) |  |
|  | Caregiver's occupation level | |  |  | 0.750 |
|  |  | More stable occupation | 4 (4.9%) | 1 (3.4%) |  |
|  |  | Less stable occupation | 78 (95.1%) | 28 (96.6%) |  |
|  | Monthly family income | |  |  | 0.736 |
|  |  | Below ฿10,000 ($318.82 ) | 18 (22.8%) | 6 (22.2%) |  |
|  |  | ฿10,001 - ฿30,000 ($318.82-956.48) | 46 (58.2%) | 14 (51.9%) |  |
|  |  | Above ฿30,000 ($956.48) | 15 (19.0%) | 7 (25.9%) |  |
|  | Prior experience with children | |  |  | 0.406 |
|  |  | Yes | 75 (93.8%) | 24 (88.9%) |  |
|  |  | No | 5 (6.2%) | 3 (11.1%) |  |
| Clinical parameters | | |  |  |  |
|  | Mean (SD) dmft score | | 5.94 (5.23) | 5.19 (4.61) | 0.480 ^b^ |
|  | Mean (SD) dmfs score | | 13.94 (16.27) | 10.97 (12.34) | 0.355 ^b^ |
|  | Mean (SD) VPI score | | 1.07 (0.73) | 1.02 (0.56) | 0.818 ^b^ |
| Dental health–related habits | | |  |  |  |
|  | Brushing at first tooth eruption | |  |  | 0.700 |
|  |  | Yes | 43 (52.4%) | 14 (48.3%) |  |
|  |  | No | 39 (47.6%) | 15 (51.7%) |  |
|  | Frequency of brushing | |  |  | 0.898 |
|  |  | 2 days or less / week | 4 (4.9%) | 2 (6.9%) |  |
|  |  | 3-5 days /week | 10 (12.3%) | 4 (13.8%) |  |
|  |  | 5 days or more /week | 67 (82.8%) | 23 (79.3%) |  |
|  | Frequency of snacking between meals | |  |  | 0.243 |
|  |  | Never or 1 time / day | 30 (37.0%) | 8 (27.6%) |  |
|  |  | 2 times /day | 25 (30.9%) | 414(48.3%) |  |
|  |  | 3 times or more /day | 26 (32.1%) | 7 (24.1%) |  |
| Perception related to dental caries | | |  |  |  |
|  | Perceived susceptibility (Mean (SD)) | | 17.38 (5.89) | 20.21 (8.66) | 0.117 ^b^ |
|  | Perceived severity (Mean (SD)) | | 24.51 (3.71) | 23.22 (4.71) | 0.150 ^b^ |
|  | Perceived benefits (Mean (SD)) | | 21.51 (2.83) | 21.07 (3.89) | 0.523 ^b^ |
|  | Perceived barriers (Mean (SD)) | | 12.26 (3.95) | 12.62 (4.22) | 0.697 ^b^ |
| Self efficacy to tooth brushing | | | 7.96 (1.45) | 7.48 (1.91) | 0.238 ^b^ |

^a^ χ^2^ test and ^b^ Student’s *t*-test
